# A socio-ecological approach to the determinants of animal health management: A scoping review

**DOI:** 10.1371/journal.pone.0344746

**Published:** 2026-03-20

**Authors:** Lisa Vors, Fanny Debil, Legrand Saint-Cyr, Anthony Giacomini, Nicolas Fortané, Guillaume Lhermie

**Affiliations:** 1 CIRAD, UMR ASTRE, Montpellier, France, ASTRE, CIRAD, INRAE, Univ Montpellier, Montpellier, Université de Toulouse, ENVT, Toulouse, France; 2 Direction Science sociales, Economie et Société, ANSES, Paris, France; 3 IRISSO, INRAE, Université Paris-Dauphine, Paris, France; 4 Faculty of Veterinary Medicine, University of Calgary, Calgary, Canada; 5 School of Public Policy, University of Calgary, Calgary, Canada; University of Nicolaus Copernicus in Torun, POLAND

## Abstract

With approximately 60% of human infectious diseases originating from zoonotic sources, an integrated approach to animal health management is critical. Significant barriers persist in optimizing disease control strategies, particularly regarding diagnostics, surveillance, biosecurity, and vaccination systems. Beyond technical and health-related aspects, socio-economic factors substantially influence both adoption and efficacy of intervention measures. While these determinants have been increasingly explored through integration of social sciences, such as economics, sociology, political science, into veterinary public health, most research remains confined to individual-level assessments focusing on epidemiological aspects and behavioral determinants, often neglecting broader social dynamics governing decision-making processes. Thus, this study develops a novel typology of determinants affecting the adoption of disease management measures using the socio-ecological model framework. We refer to these as “applicability factors”, defined as elements that either facilitate or hinder the implementation of animal health management measures. A scoping review was conducted to refine the classification of these factors, leading to the identification of 22,683 articles, of which 593 were analyzed. The scoping review was performed in accordance with the methodology defined by the PRISMA-ScR guidelines. Five key factors emerged: (1) individual and socio-cognitive, (2) socio-political and institutional, (3) economic, (4) organizational and professional, and (5) infrastructural. Our findings highlight critical barriers and facilitators while identifying research biases, including a predominant focus on zoonotic diseases and those with significant economic impact. The overrepresentation of studies from high-income countries underscores an imbalance in research efforts. Most studies emphasize individual-level determinants using epidemiological approaches, creating a notable gap in addressing systemic influences. Framing animal disease management within a socio-ecological model demonstrates the necessity of integrating these determinants into policy development. A systems-based approach proves essential for strengthening One Health governance. However, the current absence of cohesive and equitable global governance structures hampers strategy effectiveness. This calls for the implementation of intelligent governance mechanisms at local and global levels, coupled with appropriate tools and infrastructure to enhance disease management efficacy. The study provides a comprehensive framework for addressing multi-level determinants in animal health policy and practice.

## Introduction

Animal diseases, whether affecting livestock, wildlife, or companion animals, pose complex challenges at different scales. Beyond their direct impact on animal health, these diseases can lead to significant economic losses for agricultural producers and also affect the animal health industry, tourism, and even international trade by restricting exchanges [1,2]. Moreover, some of these diseases are transmissible to humans (zoonoses), thereby increasing global health risks and underscoring the importance of adopting a “One Health” approach that integrates human, animal, and environmental health [3].

The challenges posed by animal diseases are intensifying due to phenomena such as globalization, increasing urbanization, antimicrobial resistance, deforestation, climate change, and the fragmentation of natural habitats [4]. These factors promote the spread of animal diseases both within livestock systems and among wildlife, increasing the risk of emerging zoonoses. The growing interactions between humans and domestic or wild animals, particularly in expanding urban and rural environments, also heighten the risk of disease transmission [5,6]. Thus, effective management of animal diseases should not be limited to protecting livestock but should also encompass wildlife and companion animals, with a focus on public health and ecological balance. It requires a comprehensive and integrated approach involving multiple stakeholders, including farmers, veterinarians, the livestock industry (cooperatives, slaughterhouses, transport companies), wildlife managers, public authorities, and pet owners [7,8]. However, the absence of robust and equitable global governance mechanisms for animal health limits the ability to respond effectively to these challenges, compromising the management of zoonotic diseases and global health equity objectives [9,10].

Furthermore, despite various efforts, including the implementation of international regulatory frameworks, additional gaps remain in animal disease management programs. These gaps pertain to diagnostics, surveillance, biosecurity, vaccination systems, culling, epidemic management, and national and international coordination [5,11-12–]. In particular, combating animal diseases in regions with limited veterinary resources, as well as in wildlife species for which management tools are less developed, represents a considerable challenge [13]. Additionally, the increasing emergence of antimicrobial resistance (AMR) threatens both animal and human health. The intensive and sometimes inappropriate use of antibiotics in livestock systems and in domestic animals contributes to this resistance, exacerbating the difficulty of effectively treating certain infections [14,15].

Beyond technical and health-related aspects, the management of animal diseases is also influenced by socio-economic factors, which play a crucial role in the adoption and effectiveness of control measures. The behaviors, practices, beliefs, and perceptions of different stakeholders, as well as social organization and contractual relationships, whether among farmers, veterinarians, pet owners, or policymakers, can either facilitate or hinder the implementation of disease prevention and management strategies [16]. These aspects, often overlooked, must be incorporated into a global governance framework where animal health is currently relegated to the background compared to human health.

There has been a recent uptake of social sciences in veterinary public health. Animal Health Economics, through the onset of international societies and global programme such as GBADS (Global Burden of Animal Diseases) has been one of the first disciplines investigating the importance of economics for decision making in animal health management [17]. Recognizing the bounded rationality of factors, sociology and political science have also explored the challenges faced in veterinary public health. However, most research on these factors falls under the concept of “acceptability,” which can be understood as an individual’s assessment of an intervention’s relevance but may also reflect a broader collective judgment on the nature of a health intervention [18]. This concept is generally limited to incorporate in epidemiology studies, individual and collective behaviors, and their impact on the implementation of intervention measures, particularly through Knowledge, Attitudes, and Practices (KAP) studies [19]. It frequently fails to account for more complex social dynamics that influence these behaviors. These may include aspects such as power relations, cultural norms, economic inequalities, and access to resources, all of which significantly shape individuals’ perceptions and reactions to health interventions. By neglecting these dimensions, traditional approaches risk providing a fragmented understanding of the obstacles to adopting control measures, thereby limiting the effectiveness of implemented strategies. Thus, identifying and understanding these determinants is crucial not only for improving disease management but also for elucidating how broader social factors shape decision-making processes. Indeed, decision-making in animal health management results from a complex interplay of social, economic, and institutional factors that define the boundaries between public and private interests.

In this study, we used the socio-ecological model to propose a new classification of key determinants that influence the adoption of health management measures, considering local contexts and the specific dynamics of different systems (livestock, wildlife, and companion animals). We refer to these determinants as “applicability factors”, meaning the contextual and socio-economic conditions that can either enable or constrain the effective implementation of animal health management measures. To develop this typology, we conducted a scoping review of the literature. The scoping review was conducted following the guidelines established in the PRISMA-ScR statement. By refining the analysis enablers and barriers to implementation, this framework supports the design of more effective and sustainable management strategies. Furthermore, by offering concrete pathways to strengthen animal health governance, our work contributes to enhancing governance frameworks on a global scale. Given the growing recognition of the interconnections between animal and human health, our approach fosters the development of integrated health policies that are responsive to both global and local challenges.

## Materials and methods

### Adaptation of Socio-ecological model for the categorization of applicability factors

The socio-ecological model originates from the work of Bronfenbrenner [20,21] in developmental psychology, which conceptualized the individual as operating within nested systems, including the microsystem, mesosystem, exosystem, and macrosystem. This perspective highlights that individual behavior is shaped by multiple interactions between the person and the social and institutional environments in which they operate. From the 1980s–1990s, this conceptual framework was widely adopted and adapted in public health, notably by McLeroy et al. [22], to better understand the determinants of health behaviors. The model postulates that different levels of influence, individual (knowledge, attitudes, perceptions), interpersonal (social networks, collective norms), organizational (institutions, professional practices), community and infrastructural (available resources, facilities), and sociopolitical (public policies, regulations, governance), interact to shape the adoption or rejection of preventive and management measures. This approach goes beyond an exclusively individual-focused perspective, highlighting structural and systemic determinants of health inequalities [23,24]. It has been successfully applied to numerous public health issues, such as tobacco prevention, physical activity, vaccination, mental health, and emerging infectious disease prevention [25,26].

In the context of animal health, the decision-making processes that influence the applicability and effectiveness of management measures (diagnosis, vaccination, biosecurity, surveillance) are similarly subject to multifactorial and intertwined influences. However, most studies focus on individual or epidemiological determinants, neglecting institutional, economic, organizational, or infrastructural factors. To address this gap, we drew on the socio-ecological model to propose a classification adapted to the veterinary context. This adaptation was carried out in two stages. First, we used and adapted the socio-ecological framework to propose five categories of factors relevant to animal health, preserving the multi-level and interactive nature of the original model. This process resulted in five main categories: individual and socio-cognitive, socio-political and institutional, economic, organizational and professional, and infrastructural factors. Individual level became individual and socio-cognitive factors, capturing knowledge, perceptions, beliefs, and socio-demographic traits of stakeholders such as farmers or veterinarians. The interpersonal and organizational levels were regrouped as organizational and professional factors, emphasizing the role of professional networks, veterinary services, and stakeholder interactions that are central to animal health governance. The community/environmental level was reframed as infrastructural factors, to account for the tangible resources, diagnostic laboratories, clinics, quarantine facilities, farm structures, on which the feasibility of measures often depends. The societal/policy level was adapted into socio-political and institutional factors, reflecting the critical role of regulations, public policies, governance structures, and institutional coordination. Finally, we introduced economic factors as a distinct category. While economic constraints are transversal to all levels of the original model, in the veterinary context they have emerged as a major determinant of applicability, encompassing costs of interventions, compensation schemes, and resource availability. Second, we used the framework to conduct the scoping review, detailed in the following section, to consolidate this typology, assess its empirical relevance, and illustrate it using determinants actually studied in the corpus of articles.

### Scoping review

A scoping review of the literature was conducted to identify available animal disease management methods and highlight the factors that may hinder or facilitate their implementation. This method enables a transparent collection of evidence on a given research topic and lies midway between narrative reviews and systematic reviews. It allows for an assessment of the breadth of research conducted in a specific field or subject by identifying relevant publications and knowledge gaps. Thus, conducting a scoping review facilitates a comprehensive state-of-the-art analysis on a research topic and constitutes a structured and analytical inventory of scientific literature on the subject. The review was carried out following the steps identified by Arksey and O’Malley (2005) [27], as well as the concepts developed by Levac et al. (2010) [28], Colquhoun et al. (2014) [29], and the methodology outlined by Tricco et al. (2018) [30]. The methodological approach developed in this study was conducted in accordance with the PRISMA-ScR checklist, which is provided in the supplementary materials. This review was guided by the following research question: “What are the factors influencing the applicability of animal disease management measures?”, with the objective of providing a typology of applicability factors for animal health management measures.

#### Data collection.

The literature search was conducted in two major databases, Web of Science and Scopus, selected for their extensive coverage of scientific fields and disciplines. Several relevant keywords related to disease management were selected and used to perform searches within these databases. The keywords were combined into queries using Boolean operators and searched in the titles, abstracts, and keywords of publications to maximize the number of results obtained. The selected keywords and the queries tailored to the specific requirements of each database are available in Supporting information. For this review, the search was restricted to peer-reviewed research articles in English and French, with no geographical limitations. The search covered the period from January 2010 to April 2025. The citations of the retrieved publications were imported into Zotero and subsequently into Excel (Version 2019) for the selection of articles and data extraction.

#### De-duplication.

The citations of the articles extracted from both databases were compiled into a single Excel file. Manual duplicate removal was then performed by a single reviewer, ensuring that each retained article was unique.

#### Eligibility screening.

The selection of articles was carried out in three phases, with the definition of eligibility criteria that were evaluated, modified, and approved by all reviewers (six in total). In the first phase, an initial screening was conducted based on the titles of the articles, each assessed by two reviewers. Given the large number of articles, the collection was subdivided to exclude those pertaining to technical disciplines, such as vaccine biology and diagnostic tests. In the second phase, the articles were screened through abstract reading, also performed by two reviewers. At this stage, new inclusion and exclusion criteria were established: the articles had to provide information on the management measures under study and specify how these measures were implemented. Finally, the articles deemed eligible for the study underwent a full-text analysis in the third phase. At this stage, the articles were divided into three collections, with two reviewers assigned per collection. Additional eligibility criteria were introduced: the articles had to present factors influencing the applicability of animal disease management measures. Applicability factors refer to elements that may influence the effective implementation of management strategies, including intervention, control, or eradication measures for animal diseases. At each stage, articles under dispute were resolved through discussion among reviewers. For the final phase, in order to obtain a comprehensive overview of the applicability factors examined in the literature, an article was included if at least one of the two reviewers supported its inclusion. The complete set of eligibility criteria applied at each stage is available in S5 Table.

#### Data extraction.

A data extraction form was developed by all authors using Microsoft Excel (Version 2019). The information collected included the article references (authors, title, year of publication), a general description (studied country, sector or species, central focus of the study, disease under investigation, scale of analysis), the context and impact of study implementation, the management measures and strategies employed, as well as the various applicability factors. All steps involved in the selection of articles were carried out following the PRISMA method [30].

#### Data analysis.

The data were recorded and organized using Excel software. Descriptive statistical analyses were performed using RStudio (version 4.2.2, 2022-10-31). These analyses included the calculation of frequencies for various variables, such as country, level of analysis, studied species, targeted diseases, and management measures. In addition, a textual analysis was conducted on the content of the extraction grid (i.e., the standardized fields synthesizing information from each article), rather than on the full texts of the articles. The aim of this analysis was to identify the most frequently used terms within each category of applicability factors and to support the identification of recurring conceptual patterns in the literature. This analysis was inspired by approaches from lexical analysis, lexicometry, and semantic field studies, which provide a structured way to detect recurring terms, conceptual patterns, and the semantic relationships between words within each factor category [31–33]. Lexical frequency analysis was performed using RStudio (tidyverse and tidytext packages). The preprocessing workflow included removal of punctuation, numbers, and stopwords (common words such as the, and, of that carry little analytical value), followed by tokenization (splitting text into individual words) and lemmatization (reducing words to their base or root form). Applied to the extraction grid, this procedure complemented the descriptive statistics by highlighting dominant terms and semantic regularities within each factor category, thereby contributing to the identification of patterns in how applicability factors are reported in the literature.

## Results

### Article search and selection

**Fig 1:**
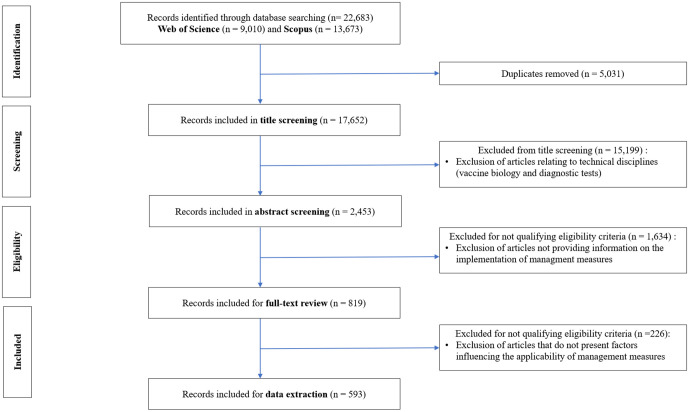
PRISMA diagram.

The search conducted in the Web of Science and Scopus databases yielded a total of 22,683 articles (9,010 from WOS and 13,673 from Scopus). The removal of duplicates reduced the number of selected articles to 17,652. The screening phase, based on title review, led to the exclusion of 15,199 articles, while the abstract review resulted in the exclusion of 1,634 articles. A total of 819 articles were retained for full-text review. Following this final selection step, 593 articles were included for analysis in this study (Fig 1). The articles were published between 2010 and 2025. Our results indicate a growing number of publications over this period, with a peak of interest in 2024 (12%) and 2020 (9%) (see S6 Table and S1 Fig).

### General characteristics of the selected articles

#### Geographical scope.

**Fig 2:**
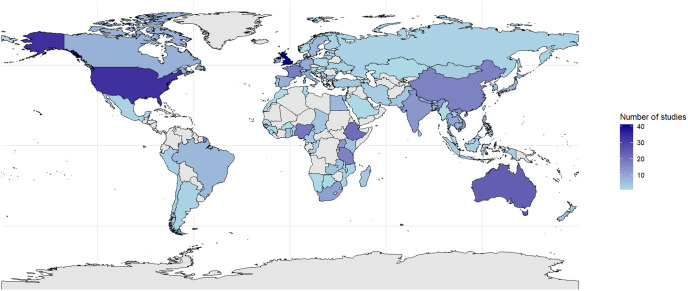
Distribution of studies by country. Eight studies in Africa, 4 studies in Latin America, 5 studies in Asia, 1 study in developed countries, 1 study in developing, countries 12 studies in Europe, 13 studies worldwide, 3 studies in LMICs, 1 study in the Mediterranean Basin, 1 study in North Africa and the Middle East, 1 study in Sub-Saharan Africa, 1 study in South America, 1 study in South Countries, 1 study in East Africa, 1 study in Caribbean region, 1 study in Asia-Oceania countries and 2 studies in South East Asia. The figure was generated using the rnaturalearth package in RStudio.

The 593 articles included in this study cover data from 91 countries (Fig 2). Most studies were conducted in high-income countries, particularly the United Kingdom (7%), Australia (4%), the United States (6%), China (3%), and France (3%). Some research stemmed from low- and middle-income countries, such as Nigeria (3%), Ethiopia (4%), Tanzania (3%), and India (2%). Most of these studies were conducted at the national level (24%), followed by farm-level (15%), regional (17%), local (16%), and individual-level (8%) studies. Additionally, some studies were conducted at broader geographic scales, with 8 studies focusing on the African continent**,** 12 on Europe**,** and 5 on Asia (see S7 Table).

#### Academic disciplines.

The disciplines involved in the studies identified were mainly epidemiology (29%), social sciences (32%), economics (19%), and life sciences (12%) (Table 1). Included studies were predominantly qualitative (31%), with a strong representation of survey-based research (21%). Quantitative analyses (21%) were also common, including modeling studies (5%) (Table 1). Furthermore, the majority of studies (67%) were conducted in routine contexts, outside of emergency situations. (Table 1) The complete characteristics of the studies are available in S8 Table.

**Table 1:** Summary of the five most represented among included studies.

Study Characteristics	Number Of Studies (Percentage)
**Type Of Study**	
Qualitative	187 (31%)
Survey	122 (21%)
Quantitative	125 (21%)
Quantitative And Qualitative	49 (8%)
Evaluation	44 (7%)
Modeling	30 (5%)
**Disciplines**	
Epidemiology	159 (27%)
Social Sciences	192 (32%)
Economics	114 (19%)
Life Sciences	73 (12%)
Psychology	22 (4%)
**Situation**	
Routine	395 (67%)
Crisis	124 (21%)
Routine And Crisis	17 (3%)
**Animal Disease**	
Rabies	146 (25%)
Avian Influenza	99 (17%)
Bovine Tuberculosis	68 (11%)
Fmd	63 (11%)
African Swine Fever	49 (8%)
**Species**	
Cattle	140 (24%)
Dog	129 (22%)
Poultry	117 (20%)
Pig	97 (16%)
Equine	23 (4%)
**Management Measures**	
Vaccination	234 (39%)
Biosecurity	191 (32%)
Surveillance	133 (22%)
Control	73 (12%)
Culling	56 (9%)

#### Disease coverage.

Included studies provide data on 53 animal diseases. Among the most studied diseases, rabies is the most represented, with 146 identified studies (25%), followed by avian influenza (17%), bovine tuberculosis (11%), foot-and-mouth disease (11%), and African swine fever (8%) (Table 1). Regarding studied species, cattle are the most represented (24%), followed by dogs (22%), poultry (20%), and pigs (16%). This distribution reflects the prevalence of the targeted diseases in our dataset (Table 1). Some studies also focus on wildlife species, such as badgers (1%), wild boars (2%), and foxes (0.7%), which play a key role in disease transmission, as seen with wild boars and African swine fever. However, the majority of research remains centered on livestock species (69% of the dataset). Detailed information on the diseases and species represented in our study is available in S8 Table**.**

#### Animal health interventions.

In our study corpus, vaccination emerged as the most extensively studied management measure, identified in 234 out of 593 studies (39%) (Table 1), followed by biosecurity strategies (32%) and surveillance programs (22%). Regarding the stakeholders involved, farmers were the most represented, accounting for 51% of the studies, followed by citizens (11%), veterinarians (11%), and dog owners (8%) (Table 1). A complete list of management measures and the stakeholders involved is available in S9, S10 Tables and S2, S3 Figs.

### Factors influencing the applicability of animal health management measures

Using the theoretical framework of the socio-ecological system, we established five key categories of factors that influence the implementation of animal health management measures. These categories allowed us to classify the relevant articles from the literature. The five categories include: (1) individual and socio-cognitive factors, (2) organizational and professional factors, (3) economic factors, (4) socio-political and institutional factors, and (5) infrastructural factors. This new classification is based on the premise that these factors must be considered interactively to fully understand the application of animal health management measures. Indeed, their implementation depends on the continuous interplay of these factors and their influence on decision-making by the various stakeholders involved. Data extracted from the literature highlighted the predominant role of individual and socio-cognitive factors (56%), underscoring the crucial importance of stakeholders’ perceptions, knowledge, and attitudes in the adoption of management measures (see S11 Table).

**Fig 3:**
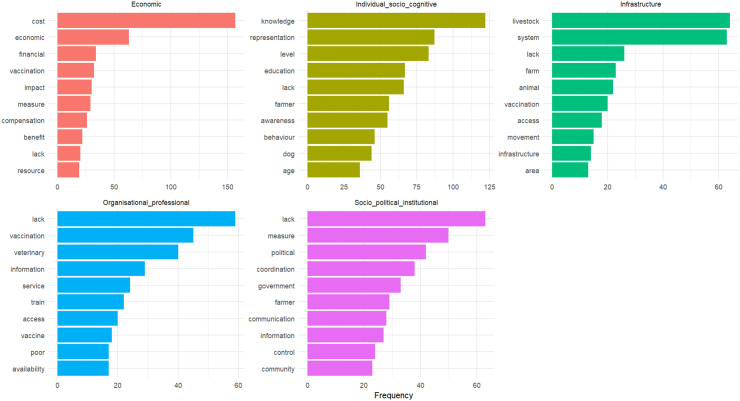
Most frequent terms identified through lexical analysis of the extrication grid by applicability factors. **A)** Top 10 words for economic factors; **B)** Top 10 words for individual and socio-cognitive factors; **C)** Top 10 most frequent words for infrastructural factors; **D)** Top 10 words for organizational and professional factors; **E)** Top 10 words for socio-political and institutional factors.

Fig 3 illustrates the most frequently mentioned terms identified through lexical analysis of the extraction grid for each factor. These terms derive from the standardized information recorded during data extraction process (see S12 Table). A detailed description of each factor is provided in the following sections to offer a comprehensive understanding of their role in animal health management. The frequency analysis is used here as an exploratory and illustrative tool to visually and systematically highlight recurring themes across a heterogeneous body of literature, rather than to infer quantitative importance. In this respect, it complements the descriptive statistical results by revealing how applicability factors are most commonly framed and discussed in the reviewed studies. Similar approaches have been applied in veterinary science, where text mining methods have been used to extract and synthesize recurring patterns from diverse sources, including online forums [34] and veterinary clinical records [35].

#### Individual and socio-cognitive factors.

Individual and socio-cognitive factors refer to the set of personal characteristics, knowledge, beliefs, attitudes, and perceptions that shape stakeholders’ understanding and decision-making regarding animal health management measures. This category encompasses both cognitive elements, such as disease knowledge and risk perception, and socio-demographic attributes, including age, gender, education level, farming experience, and household characteristics, which influence how individuals interpret information and act upon it. The most frequently mentioned terms were “knowledge,” “representation,” “level,” and “education.” Studies consistently show that stakeholders’ knowledge and awareness determine their recognition of the importance of preventive and control measures, such as vaccination and biosecurity protocols [36,37]. Past experiences and cultural traditions often shape attitudes and beliefs, leading some livestock farmers to favor traditional practices over modern veterinary interventions [38,39]. Trust in authorities and veterinary professionals is a key determinant, with higher trust facilitating compliance and skepticism limiting adoption [40]. Additionally, risk perception and risk aversion influence prioritization of preventive versus reactive measures [41], while social dynamics, including peer influence and fear of judgment, further affect individual decision-making [42]. Together, these factors critically shape the adoption and consistency of management measures, highlighting the importance of understanding individual knowledge, attitudes, perceptions, and socio-demographic characteristics when designing effective disease control strategies.

#### Organizational and profession factors.

Organizational and professional factors are defined as the characteristics of the structures, practices, and capacities of the organizations and professionals involved in animal health, including veterinary services, professional associations, and farmers’ networks. This category encompasses aspects such as coordination between stakeholders, availability and quality of professional support, access to trained personnel, and adequacy of equipment and resources. The most frequently cited terms were “lack,” “vaccination,” “veterinary,” “information,” and “service.” Numerous studies emphasize that effective interactions among stakeholders, particularly between farmers and veterinarians or technical support systems, are essential for the timely dissemination of information and the implementation of best practices [43]. A lack of coordination and communication between these actors has often been reported as a barrier to early disease detection and efficient management [44]. In addition, limited capacity of veterinary services, poor-quality provision, shortages of qualified personnel, and insufficient equipment further constrain the ability to respond to health crises effectively [45,46]. Recent global mapping of access to veterinary services further illustrates how uneven distribution of veterinary practices and limited availability of trained personnel create significant barriers, particularly in low- and middle-income countries, while also highlighting the strong variability of these organizational and professional determinants across contexts [47]. These findings appear across multiple contexts and countries, highlighting that organizational and professional factor represent systemic determinants with broad implications for the adoption and success of disease management measures.

#### Economic factors.

For economic factors, the key terms were “cost,” “economic,” “impact,” “financial,” and “vaccination.” These factors primarily relate to the costs associated with management measures, financial losses due to animal diseases, trade and market impacts, economic incentives, and financial constraints. The cost of implementing management measures is a major factor influencing their feasibility, as it is largely borne by farmers, who often perceive the return on investment as uncertain [48,49]. Financial losses linked to the adoption of these measures discourage farmers from applying them, especially when no financial compensation is provided by governments [50]. Studies have shown that demonstrating the economic benefits of implementing management measures, along with offering financial compensation, could encourage farmers to adopt them [51]. Furthermore, research has highlighted a lack of dedicated funding for animal disease management, particularly in resource-limited countries, which hinders the effective implementation of necessary measures [52,53].

#### Socio-political and institutional factors.

In the socio-political and institutional category, the most common words were “measure,” “political,” “lack,” “coordination,” and “government.” These factors encompassing policies, regulations, and institutional frameworks that influence animal disease management. A lack of communication and coordination among stakeholders, who often have diverging interests, particularly between public health authorities and veterinary services, has been widely documented [54]. Establishing entities that facilitate dialogue while integrating scientific knowledge and local needs is essential to ensure that community-specific factors are considered in decision-making processes [55]. This approach can enhance the acceptance and implementation of management measures. Decentralization and subsidiarity have emerged in several studies as potential solutions to address local specificities and improve information dissemination [56]. Additionally, political instability and armed conflicts can further hinder the implementation of disease management measures [48]. Public authorities play a crucial role in disseminating health regulations and raising community awareness. They must also provide essential resources, such as access to training programs, to strengthen animal disease management strategies [57].

#### Infrastructural factors.

Finally, for infrastructural factors, the most prevalent terms were “system,” “livestock,” “farm,” “lack,” and “animal.” These factors were identified as essential components of animal disease management, encompassing physical infrastructure such as veterinary clinics, quarantine facilities, laboratories, and vaccination sites. The structure of livestock farms has been documented as influencing the implementation of disease management measures. A frequent issue is the lack of space to isolate sick animals and regulate herd populations, which hampers disease control efforts [58]. Additionally, limited laboratory capacity poses a major challenge, with shortages of diagnostic tests, treatments, and even vaccines restricting effective disease management [59]. Furthermore, the limited availability of veterinary services, particularly in remote areas, makes access to animal healthcare difficult for farmers [60]. Fragmented health systems further complicate the implementation of management measures by restricting access to essential treatments and vaccines [61].

**Fig 4:**
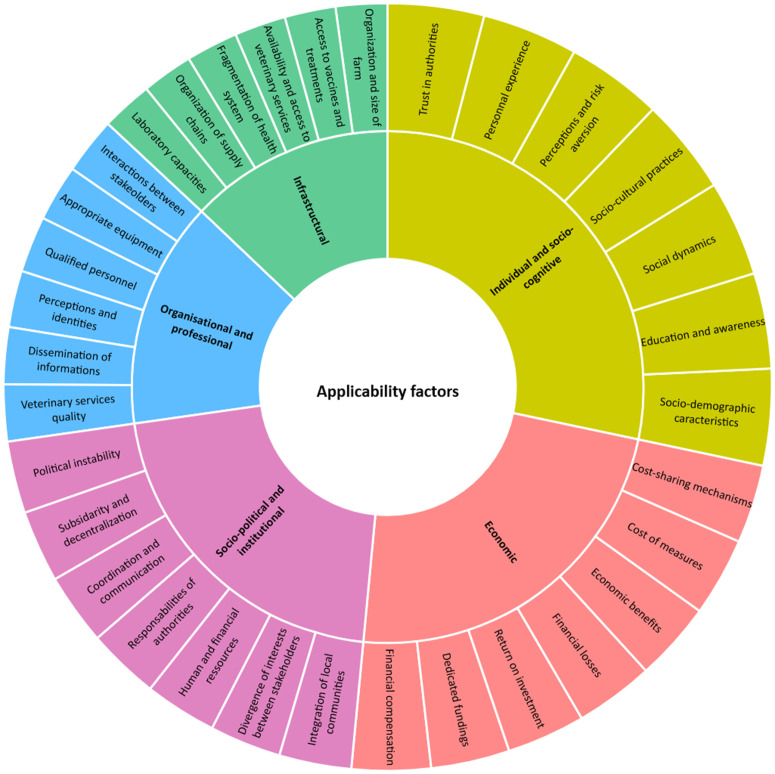
Applicability factors of animal diseases management.

Some words appear across multiple categories, such as the term “lack,” which is found in every category, and “vaccination,” which is mentioned in the infrastructural, economic, and organizational and professional factors. This highlights the value of a multifactorial approach, underscoring the complex and interconnected nature of the factors that influence the implementation of animal health management measures. Fig 4 illustrates all the factors defined in this study and their relative significance within our corpus.

## Discussion

Non-technical factors play a pivotal role in the implementation of any policy. Individual and collective behaviors, along with decision-making processes, directly influence the effectiveness of interventions. These behaviors are shaped by determinants that interact across multiple levels [23,24]. To analyze how these factors affect stakeholders’ decision-making in animal health, particularly regarding the implementation of disease management measures, we used the socio-ecological model as our theoretical framework. Originally developed in developmental psychology [20] and later widely applied in public health [22], this model emphasizes that individual behaviours are embedded within, and shaped by, multiple interacting levels of influence, from individual cognition to institutional, infrastructural, and policy contexts. Building on this foundation, we defined categories of applicability factors tailored to the veterinary field, incorporating dimensions such as veterinary services, professional practices, and infrastructures as distinct components. This adaptation was conducted prior to data encoding and subsequently consolidated through our scoping review, which allowed us to test the relevance of this framework against a heterogeneous body of literature. By combining a robust conceptual model with empirical validation, we introduced a classification of applicability factors that either facilitate or hinder the adoption of management measures. Importantly, this approach moves beyond a compartmentalized reading of determinants: the factors we identified do not operate in isolation but interact dynamically across levels. Overlooking these interactions risks oversimplifying the complexity of animal health governance and underestimating systemic barriers to effective disease management. Our contribution therefore lies in providing both a novel analytical lens and an empirically grounded typology to guide the study of applicability factors in animal health. This integrated perspective highlights the need for context-specific, multi-level interventions that recognize systemic interdependencies and can support more coordinated and equitable governance within a One Health framework.

A scoping review was conducted, identifying 593 articles included in this study. We chose this method to allow for a broad examination of available studies, in contrast to more traditional systematic reviews, such as meta-analyses. Additionally, this approach is useful for identifying knowledge gaps and mapping evidence on a given topic [29]. However, while this method enabled us to include a wide variety of studies, it may also have resulted in variability in the methodological rigor of the selected research, with some studies being less robust than others. Furthermore, our selection was limited to texts in English and French, which may have excluded relevant research published in other languages. This could introduce a language bias and limit the diversity of perspectives on the studied topic. We also chose to restrict the data extraction period to studies published between 2010 and 2025, allowing us to focus on recent work. However, this decision excluded older studies that might have provided important historical context or additional insights. Finally, the categories of applicability factors were defined before data extraction, based on the principles of socio-ecological model. While this approach allowed for a structured and coherent analysis, it may have introduced a selection bias by influencing how we interpreted and classified study results. A more inductive approach could have facilitated the identification of unanticipated applicability factors.

Our analysis reveals a strong overrepresentation of studies from developed countries, particularly economic powers such as the United Kingdom, the United States, Australia, China, and France. This trend reflects their substantial financial, institutional, and scientific resources, facilitating research on animal disease management [11]. Additionally, their structured livestock industries necessitate robust health control strategies to maintain productivity and trade relations [62,63]. Consequently, intensive livestock systems dominate the literature, while extensive systems, more common in the Global South, remain underrepresented [63]. Developing countries, despite their high exposure to health risks, face limited research due to budget priorities favoring human health and a lack of veterinary infrastructure and surveillance programs [13]. This imbalance highlights gaps in knowledge on local constraints and solutions tailored to low-resource settings. Strengthening international cooperation and investing in data collection are crucial to enhancing disease control and pandemic preparedness, especially within the One Health framework, which recognizes the interdependence of animal, human, and environmental health.

Our results also highlight an overrepresentation of certain diseases, rabies, avian influenza, bovine tuberculosis, and foot-and-mouth disease, compared to others such as brucellosis or salmonellosis. This bias is largely driven by economic and health impacts, trade implications, and zoonotic risks [64,65]. For instance, avian influenza and foot-and-mouth disease impose heavy economic losses due to movement restrictions and culling measures [66,67]. Rabies and bovine tuberculosis, given their zoonotic potential, are prioritized in international programs, such as the 2015 WHO-OIE-FAO-GARC initiative to eliminate rabies by 2030 [68]. Additionally, livestock species receive significantly more research attention than companion animals, aquaculture, or beekeeping, which are often overlooked as a form of “undone science” [69], reflecting the economic dominance of the livestock industry [70]. Neglecting these underrepresented species and diseases may pose future risks, particularly as climate change and globalization alter disease dynamics. Additionally, vaccination emerges as the most studied disease management strategy in our corpus, widely recognized for its role in controlling infectious diseases, as seen in the eradication of rinderpest and smallpox [71]. Vaccination campaigns, particularly for zoonoses like rabies, are crucial, yet their implementation faces challenges. Resistance from farmers and consumers, ethical concerns regarding animal testing, and the financial burden of large-scale campaigns remain key obstacles [71]. Furthermore, serological interference in vaccinated animals can lead to trade restrictions, as observed with avian influenza and foot-and-mouth disease [71]. Our findings also underscore the central role of farmers in disease management, while other key stakeholders, veterinarians, health authorities, and policymakers, appear less involved in implementing control measures. Effective disease control requires coordinated action from all actors, including researchers, international organizations, and consumers [72,73]. However, structural barriers such as weak regulatory frameworks, resource constraints, and limited veterinary access hinder broader engagement [74]. Addressing these challenges is crucial for a more inclusive and effective approach to animal health governance.

This study proposes a new typology of applicability factors influencing animal disease management measures. We identified five categories, reinterpreting the socio-ecological model to integrate key dimensions such as power dynamics, resource access inequalities, and the bidirectional nature of influences across levels as shown in Fig 4. This approach moves beyond a simplistic behavioral perspective, providing a more nuanced understanding of the facilitators and barriers to disease management. Rather than adhering to the traditional nested and hierarchical structure of the socio-ecological model [75], commonly used in public health, we emphasize the complex interplay between these factors. We identified individual and socio-cognitive factors that shape the adoption of management measures, particularly through actors’ knowledge and perceptions of health risks [41]. While awareness initiatives improve adoption [76], ignorance, erroneous beliefs, and cultural resistance can hinder implementation [39–77]. These factors are often analyzed using the Knowledge, Attitudes, and Practices (KAP) approach, which tends to be reductionist by focusing primarily on individual behavior changes while overlooking structural constraints and power dynamics. As shown in Fig 3, which presents the most frequently occurring words for each factor, terms such as knowledge, representation, education, awareness, behavior, practice, and age are the most prominent in this category, perfectly illustrating the prevalence of KAP-based studies. However, actors operate within institutional and regulatory frameworks where political and industrial interests may not align with their needs [78,79]. Moreover, such models often assume a one-way influence of policies on behaviors, overlooking feedback loops and the capacity of local practices to reshape governance frameworks. By ignoring socio-economic inequalities, they risk masking mechanisms of marginalization that restrict access to veterinary resources and services, further complicating effective disease management [80].

Thus, we also identified organizational and professional factors, particularly the availability and quality of veterinary services, as well as interactions between stakeholders, such as farmers, peers, and health or technical advisors. Effective coordination among these actors is crucial to ensuring seamless information transfer and fostering the adoption of management measures. Strengthening veterinary services, through improved infrastructure, equipment, and staff training, is essential to enhancing disease control efforts. Additionally, we emphasized the critical role of economic factors, particularly the costs associated with management measures and the availability of resources for their implementation. This is especially relevant for farmers, who may be reluctant to adopt such measures if they do not perceive immediate benefits. Financial mechanisms, such as compensation, have been shown to facilitate adoption [50]. This is further illustrated in Fig 3, where the most frequently occurring terms in this category include cost, economic, impact, financial, and compensation. However, it is crucial to go beyond simple cost-benefit analysis by considering socio-economic inequalities that may obscure marginalization mechanisms, preventing certain groups from accessing the necessary resources and veterinary services for effective disease management. These factors are also linked to socio-political and institutional factors, which emphasize the importance of clear norms and policies to facilitate the adoption of measures. Coordination among institutional actors is essential, as divergences or lack of communication between these entities can hinder the effective implementation of measures [53]. Therefore, incentive policies and collaborative governance mechanisms could enhance the implementation and execution of disease management interventions. Finally, understanding the role of infrastructural factors is crucial. The reviewed studies highlight the impact of equipment availability, such as diagnostic tests, storage facilities for medicines and vaccines, and access to new technologies, on the adoption of management measures [81]. Conversely, inadequate infrastructure can be a major barrier, particularly when limited farm space prevents the isolation of diseased animals [59]. Additionally, the geographical isolation of certain farmers restricts access to veterinary services, diagnostic tools, and treatments, further complicating disease management [60].

Building on the socio-ecological model, it is important to emphasize that the five categories of applicability factors do not operate in isolation. Individual and socio-cognitive factors are influenced by, and in turn influence, organizational, economic, institutional, and infrastructural contexts, creating a network of interdependent determinants. For example, a farmer’s knowledge and perception of disease risks may shape their adoption of vaccination, but these behaviors are constrained or facilitated by the availability and quality of veterinary services, access to financial resources, and supportive institutional policies. Similarly, institutional regulations and governance structures can enable or restrict professional practices and resource distribution, which subsequently affect individual-level behaviors. By framing animal health management within this interactive socio-ecological lens, we highlight the bidirectional and feedback relationships across levels: interventions at the policy or organizational level can modify individual behaviors, while collective experiences and local practices can, over time, influence institutional and governance frameworks. Recognizing these interdependencies underscores the need for integrated, multi-level strategies that align behavioral, organizational, economic, and infrastructural levers, rather than isolated interventions, to effectively enhance the adoption and implementation of disease management measures.

These findings highlight the necessity of a multifactorial approach to animal disease management, ensuring that key factors are integrated into decision-making rather than treated as external constraints (Fig 4). Neglecting this complexity risks leading to reductionist strategies that overlook the diverse actors and interactions involved, ultimately undermining the effectiveness and sustainability of disease control efforts. Our classification of applicability factors underscores the critical role of information access and dissemination in shaping the adoption of management measures. Effective peer communication fosters knowledge-sharing and collective action, while the spread of information, whether accurate or misleading, can significantly influence adherence to health measures, particularly in the digital age. While social media can enhance awareness, it also facilitates misinformation, eroding trust in authorities and encouraging risky behaviors [82]. This was evident during the COVID-19 pandemic, where conspiracy theories fueled vaccine hesitancy and resistance to public health measures [83]. Similar challenges arise in animal health, particularly regarding vaccine safety, treatment efficacy, and biosecurity compliance. To address these issues, health authorities must adopt proactive communication strategies that ensure transparent and coherent dissemination of reliable information. Integrating these elements into disease management strategies is essential for improving compliance and health outcomes. A robust governance framework, built on coordinated policies, interdisciplinary collaboration, and effective risk communication, can strengthen disease control efforts and enhance the resilience of animal health systems.

## Conclusion

In conclusion, the proposed framework constitutes an initial step toward systematically integrating multi-level and interdependent determinants into animal health management, with future applications expected to refine its categories and demonstrate their interactions in practice. This research highlights several key points that resonate with the global challenges we face today in public health, particularly in the context of global health governance. Global health relies on a systemic approach, emphasized by the One Health approach, which recognizes the interdependence of human, animal, and environmental health factors. This perspective is particularly relevant when analyzing the factors that influence the adoption of animal disease management measures. Indeed, these factors are not limited to veterinary or zootechnical aspects alone but are also shaped by social, political, economic, and organizational dimensions. All of the factors identified in this review are part of a broader dynamic of global health, where the management of animal diseases cannot be separated from human health management. In an era where health challenges are increasingly global, it is essential to adopt a One Health approach that recognizes the interconnection between these different spheres of health. Such an approach will help better understand and overcome the obstacles to adopting more sustainable and suitable management measures. Integrated public policies that consider the economic, social, and infrastructural realities of animal disease management systems are essential. Interventions tailored to the local level aimed at improving stakeholder perceptions are necessary to facilitate the adoption of management measures.

## Supporting information

S1 Table Keywords selected for articles search. (DOCX)

S2 Table Search engines and queries. (DOCX)

S3 Table Subject area selected by searching articles in Scopus. (DOCX)

S4 Table Web of Science categories selected by searching articles in Web of Sciences. (DOCX)

S5 Table Summary of eligibility criteria for selection of articles included in the review. (DOCX)

S6 Table Frequency of studies by year. (DOCX)

S7 Table Frequency of studies by country. (DOCX)

S8 Table Characteristics of reviewed studies. (DOCX)

S9 Table Representation of management measures. (DOCX)

S10 Table Actors involved in animal diseases management. (DOCX)

S11 Table Frequency of applicability Factors. (DOCX)

S12 Table Most frequent word for each applicability factors. (DOCX)

S13 Table Scoping reviews (PRISMA-ScR) Checklist. (DOCX)

S14 Table Final articles included in the study and data extracted. (XLSX)

S1 Fig Occurrence of reviewed studies by year of publication. (DOCX)

S2 Fig Top 10 of management measures. (DOCX)

S3 Fig Top 10 of actors involved in animal diseases management. (DOCX)

S4 Fig Representation of applicability factors. (DOCX)

## References

[1] Rich KM, Perry BD. The economic and poverty impacts of animal diseases in developing countries: new roles, new demands for economics and epidemiology. Prev Vet Med. 2011;101(3–4):133–47. doi: 10.1016/j.prevetmed.2010.08.002 20828844

[2] Kappes A, Tozooneyi T, Shakil G, Railey AF, McIntyre KM, Mayberry DE. Livestock health and disease economics: a scoping review of selected literature. Frontiers in Veterinary Science. 2023;10:1168649. doi: 10.3389/fvets.2023.1168649 37795016 PMC10546065

[3] Mcelwain TF, Thumbi SM. Animal pathogens and their impact on animal health, the economy, food security, food safety and public health. Rev Sci Tech OIE. 2017;36(2):423–33. 10.20506/rst.36.2.2663 PMC6561776 30152474

[4] Klous G, Huss A, Heederik DJJ, Coutinho RA. Human-livestock contacts and their relationship to transmission of zoonotic pathogens, a systematic review of literature. One Health. 2016;2:65–76. doi: 10.1016/j.onehlt.2016.03.001 28616478 PMC5462650

[5] Perez A, AlKhamis M, Carlsson U, Brito B, Carrasco-Medanic R, Whedbee Z. Global animal disease surveillance. Spatial and Spatio-temporal Epidemiology. 2011;2(3):135–45. 22748173 10.1016/j.sste.2011.07.006 PMC7185519

[6] Morand S, McIntyre KM, Baylis M. Domesticated animals and human infectious diseases of zoonotic origins: domestication time matters. Infect Genet Evol. 2014;24:76–81. doi: 10.1016/j.meegid.2014.02.013 24642136

[7] Miller RS, Pepin KM. Board invited review: Prospects for improving management of animal disease introductions using disease-dynamic models. J Anim Sci. 2019;97(6):2291–307. doi: 10.1093/jas/skz125 30976799 PMC6541823

[8] Rabinowitz P, Conti L. Links among human health, animal health, and ecosystem health. Annu Rev Public Health. 2013;34(1):189–204. 23330700 10.1146/annurev-publhealth-031912-114426

[9] Williams MA, Wyner SN. Global Health Governance: The Major Players in the Field and Their Challenges. Am J Public Health. 2017;107(12):1848–50. doi: 10.2105/AJPH.2017.304148 29116833 PMC5678416

[10] Gostin LO, Moon S, Meier BM. Reimagining Global Health Governance in the Age of COVID-19. Am J Public Health. 2020;110(11):1615–9. 33026872 10.2105/AJPH.2020.305933 PMC7542258

[11] Charlier J, Barkema HW, Becher P, De Benedictis P, Hansson I, Hennig-Pauka I, et al. Disease control tools to secure animal and public health in a densely populated world. Lancet Planet Health. 2022;6(10):e812–24. doi: 10.1016/S2542-5196(22)00147-4 36208644

[12] Cui M, Shen B, Fu ZF, Chen H. Animal diseases and human future. Anim Dis. 2022;2(1):6. doi: 10.1186/s44149-022-00041-z 35498759 PMC9035331

[13] Lindahl JF, Mutua F, Grace D. Evaluating farm-level livestock interventions in low-income countries: a scoping review of what works, how, and why. Anim Health Res Rev. 2020;21(2):108–21. doi: 10.1017/S1466252320000146 33261710

[14] Page SW, Gautier P. Use of antimicrobial agents in livestock: -EN- -FR- Utilisation des agents antimirobiens chez les animaux d’élevage -ES- Uso de agentes antimicrobianos en el ganado. Rev Sci Tech OIE. 2012;31(1):145–88. 10.20506/rst.31.1.2106 22849274

[15] Magnusson U, Moodley A, Osbjer K. Antimicrobial resistance at the livestock–human interface: implications for veterinary services. Rev Sci Tech OIE. 2021;40(2):511–21. 10.20506/rst.40.2.3241 34542097

[16] Card C, Epp T, Lem M. Exploring the Social Determinants of Animal Health. J Vet Med Educ. 2018;45(4):437–47. doi: 10.3138/jvme.0317-047r 30285599

[17] Rushton J, Bruce M, Bellet C, Torgerson P, Shaw A, Marsh T, et al. Initiation of Global Burden of Animal Diseases Programme. The Lancet. 2018 Aug;392(10147):538–40. 30152376 10.1016/S0140-6736(18)31472-7

[18] Sekhon M, Cartwright M, Francis JJ. Acceptability of healthcare interventions: an overview of reviews and development of a theoretical framework. BMC Health Serv Res. 2017;17(1):88. doi: 10.1186/s12913-017-2031-8 28126032 PMC5267473

[19] Suwannarong K, Kanthawee P, Thammasutti K, Ponlap T, Klinnoi A, Lantican C, et al. A qualitative study on knowledge, attitude, and practice (KAP) toward swine influenza, information on pig farms and zoonosis reporting systems in Thailand. Prev Vet Med. 2023;219:106020. doi: 10.1016/j.prevetmed.2023.106020 37696206

[20] Tudge J, Rosa EM. Bronfenbrenner’s Ecological Theory. The Encyclopedia of Child and Adolescent Development. Wiley. 2020. 1–11. doi: 10.1002/9781119171492.wecad251

[21] Henderson DX, DeCuir-Gunby J, Gill V. “It Really Takes a Village”: A Socio-Ecological Model of Resilience for Prevention Among Economically Disadvantaged Ethnic Minority Youth. J Prim Prev. 2016;37(5):469–85. doi: 10.1007/s10935-016-0446-3 27624607

[22] McLeroy KR, Bibeau D, Steckler A, Glanz K. An ecological perspective on health promotion programs. Health Educ Q. 1988;15(4):351–77. doi: 10.1177/109019818801500401 3068205

[23] Pao H n i, Jackson E, Yang T s u n g, Tsai J s y u n g, Sung WHT, Pfeiffer DU. Determinants of farmers’ biosecurity mindset: A social-ecological model using systems thinking. Frontiers in Veterinary Science. 2022;9:959934. doi: 10.3389/fvets.2022.959934 36046509 PMC9420990

[24] Reyes AT, Serafica R, Kawi J, Fudolig M, Sy F, Leyva EWA, et al. Using the Socioecological Model to Explore Barriers to Health Care Provision in Underserved Communities in the Philippines: Qualitative Study. Asian Pac Isl Nurs J. 2023;7:e45669. doi: 10.2196/45669 37606966 PMC10481217

[25] Pereira MMCE, Padez CMP, Nogueira HGdSM. Describing studies on childhood obesity determinants by Socio-Ecological Model level: a scoping review to identify gaps and provide guidance for future research. Int J Obes (Lond). 2019;43(10):1883–90. doi: 10.1038/s41366-019-0411-3 31285521

[26] Sallis JF, Owen NF. Ecological Models of Health Behavior. Health Behavior: Theory, Research, and Practice. 4 ed. United States of America: Jossey-Bass. 2008. 590.

[27] Arksey H, O’Malley L. Scoping studies: towards a methodological framework. International Journal of Social Research Methodology. 2005;8(1):19–32. doi: 10.1080/1364557032000119616

[28] Levac D, Colquhoun H, O’Brien KK. Scoping studies: advancing the methodology. Implement Sci. 2010;5:69. doi: 10.1186/1748-5908-5-69 20854677 PMC2954944

[29] Colquhoun HL, Levac D, O’Brien KK, Straus S, Tricco AC, Perrier L, et al. Scoping reviews: time for clarity in definition, methods, and reporting. J Clin Epidemiol. 2014;67(12):1291–4. doi: 10.1016/j.jclinepi.2014.03.013 25034198

[30] Tricco AC, Lillie E, Zarin W, O’Brien KK, Colquhoun H, Levac D, et al. PRISMA Extension for Scoping Reviews (PRISMA-ScR): Checklist and Explanation. Ann Intern Med. 2018;169(7):467–73. doi: 10.7326/M18-0850 30178033

[31] Compton T. Beyond the black box: Integrating lexical and semantic methods in quantitative discourse analysis with BERTopic. arXiv. https://arxiv.org/abs/2508.19099 2025. 2025 September 25.

[32] Petersen E, Potts C. Lexical Semantics with Large Language Models: A Case Study of English “break”. In: Findings of the Association for Computational Linguistics: EACL 2023, 2023. 490–511. doi: 10.18653/v1/2023.findings-eacl.36

[33] Baes N, Haslam N, Vylomova E. A Multidimensional Framework for Evaluating Lexical Semantic Change with Social Science Applications. In: Proceedings of the 62nd Annual Meeting of the Association for Computational Linguistics (Volume 1: Long Papers), Bangkok, Thailand, 2024. 1390–415. https://aclanthology.org/2024.acl-long.76

[34] Munaf S, Swingler K, Brülisauer F, O’Hare A, Gunn G, Reeves A. Text mining of veterinary forums for epidemiological surveillance supplementation. Soc Netw Anal Min. 2023;13(1):121.

[35] Davies H, Nenadic G, Alfattni G, Arguello Casteleiro M, Al Moubayed N, Farrell SO, et al. Text mining for disease surveillance in veterinary clinical data: part one, the language of veterinary clinical records and searching for words. Front Vet Sci. 2024;11:1352239. doi: 10.3389/fvets.2024.1352239 38322169 PMC10844486

[36] Hernández-Jover M, Taylor M, Holyoake P, Dhand N. Pig producers’ perceptions of the Influenza Pandemic H1N1/09 outbreak and its effect on their biosecurity practices in Australia. Prev Vet Med. 2012;106(3–4):284–94. doi: 10.1016/j.prevetmed.2012.03.008 22487168

[37] Bahiru A, Molla W, Yizengaw L, Mekonnen SA, Jemberu WT. Knowledge, attitude and practice related to rabies among residents of Amhara region, Ethiopia. Heliyon. 2022;8(11):e11366. doi: 10.1016/j.heliyon.2022.e11366 36387566 PMC9649959

[38] Arvidsson A, Fischer K, Chenais E, Kiguli J, Sternberg-Lewerin S, Ståhl K. Limitations and opportunities of smallholders’ practical knowledge when dealing with pig health issues in northern Uganda. PLoS One. 2023;18(6):e0287041. doi: 10.1371/journal.pone.0287041 37294750 PMC10256192

[39] Dione MM, Dohoo I, Ndiwa N, Poole J, Ouma E, Amia WC, et al. Impact of participatory training of smallholder pig farmers on knowledge, attitudes and practices regarding biosecurity for the control of African swine fever in Uganda. Transbound Emerg Dis. 2020;67(6):2482–93. doi: 10.1111/tbed.13587 32311216 PMC7754142

[40] Elbers ARW, Gorgievski-Duijvesteijn MJ, van der Velden PG, Loeffen WLA, Zarafshani K. A socio-psychological investigation into limitations and incentives concerning reporting a clinically suspect situation aimed at improving early detection of classical swine fever outbreaks. Vet Microbiol. 2010;142(1–2):108–18. doi: 10.1016/j.vetmic.2009.09.051 19854004

[41] Farrell P, Hunter C, Truong B, Bunning M. Control of highly pathogenic avian influenza in Quang Tri province, Vietnam: voices from the human-animal interface. RRH. 2015. 26163749

[42] Azbel-Jackson L, Heffernan C, Gunn G, Brownlie J. Exploring the role of voluntary disease schemes on UK farmer bio-security behaviours: Findings from the Norfolk-Suffolk Bovine Viral Diarrhoea control scheme. PLoS One. 2018;13(2):e0179877. doi: 10.1371/journal.pone.0179877 29432435 PMC5809011

[43] Bissong MEA, Lyombe JCN, Asongalem E, Ngamsha RB, Tendongfor N. Zoonotic diseases risk perception and infection prevention and control practices among poultry farmers in the Buea Health District, Cameroon: A one health perspective. Vet World. 2022;:2744–53. 36590116 10.14202/vetworld.2022.2744-2753 PMC9798056

[44] Azhar M, Lubis AS, Siregar ES, Alders RG, Brum E, McGrane J, et al. Participatory disease surveillance and response in Indonesia: strengthening veterinary services and empowering communities to prevent and control highly pathogenic avian influenza. Avian Dis. 2010;54(1 Suppl):749–53. doi: 10.1637/8713-031809-Reg.1 20521726

[45] Aptriana CD, Sudarnika E, Basri C. Nationally and locally-initiated One Health approach in controlling rabies in West Kalimantan, Indonesia. Vet World. 2022;:2953–61. 36718315 10.14202/vetworld.2022.2953-2961 PMC9880830

[46] Fenelon N, Dely P, Katz MA, Schaad ND, Dismer A, Moran D, et al. Knowledge, attitudes and practices regarding rabies risk in community members and healthcare professionals: Pétionville, Haiti, 2013. Epidemiol Infect. 2017;145(8):1624–34. doi: 10.1017/S0950268816003125 28290915 PMC5426290

[47] Criscuolo NG, Wang Y, Van Boeckel TP. A global map of travel time to access veterinarians. Nat Commun. 2025;16(1):5849. doi: 10.1038/s41467-025-60102-y 40595463 PMC12214892

[48] Adedeji AO, Eyarefe OD, Okonko IO, Ojezele MO, Amusan TA, Abubakar M. Why is there still rabies in Nigeria? - A review of the current and future trends in the epidemiology, prevention, treatment, control and possible elimination of rabies. 2010. https://api.semanticscholar.org/CorpusID:5087998

[49] Bedeković T, Šimić I, Krešić N, Lojkić I. Influence of different factors on the costs and benefits of oral vaccination of foxes against rabies. Zoonoses Public Health. 2019;66(5):526–32. doi: 10.1111/zph.12587 31119884

[50] Aliro T, Odongo W, Ståhl K, Dione MM, Okello DM, Masembe C, et al. Actions and perceived impact of African swine fever control measures along the smallholder pig value chain in Uganda. Trop Anim Health Prod. 2023;55(6):410. doi: 10.1007/s11250-023-03828-5 37987884 PMC10663180

[51] Cho J, Tauer LW, Schukken YH, Smith RL, Lu Z, Grohn YT. Cost‐Effective Control Strategies for Johne’s Disease in Dairy Herds. Canadian J Agri Economics. 2012;61(4):583–608. doi: 10.1111/j.1744-7976.2012.01270.x

[52] Bay V, Shirzadi MR, Jafari Sirizi M, Asl IM. Animal bites management in Northern Iran: Challenges and solutions. Heliyon. 2023;9(8):e18637. doi: 10.1016/j.heliyon.2023.e18637 37554820 PMC10404659

[53] Anderson A, Shwiff S, Gebhardt K, Ramírez AJ, Shwiff S, Kohler D, et al. Economic evaluation of vampire bat (Desmodus rotundus) rabies prevention in Mexico. Transbound Emerg Dis. 2014;61(2):140–6. doi: 10.1111/tbed.12007 22984914

[54] Maye D, Chivers C-A, Enticott G, Lenormand T, Tomlinson S. Social research to understand farmer and agricultural stakeholder attitudes towards bovine tuberculosis vaccination of cattle. Vet Rec. 2023;193(7):e3166. doi: 10.1002/vetr.3166 37339358

[55] Aengwanich W, Boonsorn T, Srikot P. Intervention to Improve Biosecurity System of Poultry Production Clusters (PPCs) in Thailand. Agriculture. 2014;4(3):231–8. doi: 10.3390/agriculture4030231

[56] Anyiam F, Lechenne M, Mindekem R, Oussigéré A, Naissengar S, Alfaroukh IO, et al. Cost-estimate and proposal for a development impact bond for canine rabies elimination by mass vaccination in Chad. Acta Trop. 2017;175:112–20. doi: 10.1016/j.actatropica.2016.11.005 27889225

[57] Djegui F, Gourlaouen M, Coetzer A, Adjin R, Tohozin R, Leopardi S, et al. Capacity Building Efforts for Rabies Diagnosis in Resource-Limited Countries in Sub-Saharan Africa: A Case Report of the Central Veterinary Laboratory in Benin (Parakou). Front Vet Sci. 2022;8:769114. doi: 10.3389/fvets.2021.769114 35118149 PMC8805029

[58] Abouelenien F, Eleisway M, Elshahawy I, Almidany S, Elsaidy N. Biosecurity practices in commercial and house hold poultry farms in the Delta region, Egypt: I-Correlation between level of biosecurity and prevalence ofpoultry mites. The Thai Journal of Veterinary Medicine. 2020;50(3):315–28. doi: 10.56808/2985-1130.3033

[59] Acharya KP, Subedi D, Wilson RT. Rabies control in South Asia requires a One Health approach. One Health. 2021;12:100215. doi: 10.1016/j.onehlt.2021.100215 33681445 PMC7907975

[60] Alhaji NB, Adeiza AM, Godwin EA, Haruna AE, Aliyu MB, Odetokun IA. An assessment of the highly pathogenic avian influenza resurgence at human-poultry-environment interface in North-central Nigeria: Sociocultural determinants and One Health implications. One Health. 2023;16:100574. doi: 10.1016/j.onehlt.2023.100574 37363241 PMC10288128

[61] Compston P, Limon G, Sangula A, Onono J, King DP, Häsler B. Understanding what shapes disease control: An historical analysis of foot-and-mouth disease in Kenya. Prev Vet Med. 2021;190:105315. doi: 10.1016/j.prevetmed.2021.105315 33735817

[62] Herrero M, Grace D, Njuki J, Johnson N, Enahoro D, Silvestri S, et al. The roles of livestock in developing countries. Animal. 2013;7 Suppl 1:3–18. doi: 10.1017/S1751731112001954 23121696

[63] Robinson TP, Thornton PK, Francesconi GN, Kruska RL, Chiozza F, Notenbaert AMO. Global livestock production systems. FAO and ILRI. 2011.

[64] Otte J, Hinrichs J, Rushton J, Roland-Holst D, Zilberman D. Impacts of avian influenza virus on animal production in developing countries. CABI Reviews. 2009;:1–18. doi: 10.1079/pavsnnr20083080

[65] Coleman PG, Fèvre EM, Cleaveland S. Estimating the public health impact of rabies. Emerg Infect Dis. 2004;10(1):140–2. 15078611 10.3201/eid1001.020744 PMC3322764

[66] Alders R, Awuni JA, Bagnol B, Farrell P, de Haan N. Impact of avian influenza on village poultry production globally. Ecohealth. 2014;11(1):63–72. doi: 10.1007/s10393-013-0867-x 24136383

[67] Knight-Jones TJD, Rushton J. The economic impacts of foot and mouth disease – what are they, how big are they and where do they occur?. Preventive Veterinary Medicine. 2013;112(3–4):161–73. 23958457 10.1016/j.prevetmed.2013.07.013 PMC3989032

[68] World Health Organization, Food and Agriculture Organization of the United Nations, World Organisation for Animal Health. Zero by 30: the global strategic plan to end human deaths from dog-mediated rabies by 2030. Geneva: World Health Organization. 2018.

[69] Frickel S, Gibbon S, Howard J, Kempner J, Ottinger G, Hess DJ. Undone Science: Charting Social Movement and Civil Society Challenges to Research Agenda Setting. Sci Technol Human Values. 2010;35(4):444–73. 10.1177/0162243909345836 PMC7041968 32099268

[70] Tomley FM, Shirley MW. Livestock infectious diseases and zoonoses. Philosophical Transactions of the Royal Society B: Biological Sciences. 2009;364(1530):2637–42. 10.1098/rstb.2009.0133 PMC2865087 19687034

[71] Schat KA. Vaccines and Vaccination Practices: Key to Sustainable Animal Production. Encyclopedia of Agriculture and Food Systems. Elsevier. 2014. 315–32. doi: 10.1016/b978-0-444-52512-3.00189-3

[72] Hayes L, Woodgate R, Rast L, Toribio J-ALML, Hernández-Jover M. Understanding animal health communication networks among smallholder livestock producers in Australia using stakeholder analysis. Prev Vet Med. 2017;144:89–101. doi: 10.1016/j.prevetmed.2017.05.026 28716209

[73] Hayes L, Manyweathers J, Maru Y, Loechel B, Kelly J, Kruger H, et al. Stakeholder mapping in animal health surveillance: A comparative assessment of networks in intensive dairy cattle and extensive sheep production in Australia. Prev Vet Med. 2021;190:105326. doi: 10.1016/j.prevetmed.2021.105326 33735818

[74] Jarvis LS, Valdes‐Donoso P. A selective review of the economic analysis of animal health management. J Agricultural Economics. 2018;69(1):201–25.

[75] Tompson AC, Chandler CIR. Addressing antibiotic use: insights from social science around the world. London: London School of Hygiene and Tropical Medicine. 2021. doi: 10.17037/PUBS.04659562

[76] Arjkumpa O, Yano T, Prakotcheo R, Sansamur C, Punyapornwithaya V. Epidemiology and National Surveillance System for Foot and Mouth Disease in Cattle in Thailand during 2008-2019. Vet Sci. 2020;7(3):99. doi: 10.3390/vetsci7030099 32722145 PMC7558286

[77] Bagale KB, Adhikari R, Acharya D. Regional variation in knowledge and practice regarding common zoonoses among livestock farmers of selective districts in Nepal. PLoS Negl Trop Dis. 2023;17(2):e0011082. doi: 10.1371/journal.pntd.0011082 36787295 PMC9928098

[78] Figuié MC. Intégrer les enjeux sociaux et dépasser le cadre des études ‘connaissances, attitudes, pratiques’: un défi majeur pour les approches interdisciplinaires. Approches interdisciplinaires en sante animale: dialogue entre sciences sociales et veterinaires. 1re ed. Editions Quae. 2024. 270.

[79] Hinchliffe S, Butcher A, Rahman MM, Guilder J, Tyler C, Verner‐Jeffreys D. Production without medicalisation: Risk practices and disease in Bangladesh aquaculture. Geographical Journal. 2020;187(1):39–50. doi: 10.1111/geoj.12371

[80] Haenssgen MJ, Charoenboon N, Zanello G, Mayxay M, Reed-Tsochas F, Jones COH, et al. Antibiotics and activity spaces: protocol of an exploratory study of behaviour, marginalisation and knowledge diffusion. BMJ Glob Health. 2018;3(2):e000621. doi: 10.1136/bmjgh-2017-000621 29629190 PMC5884330

[81] Alarcón LV, Monterubbianesi M, Perelman S, Sanguinetti HR, Perfumo CJ, Mateu E, et al. Biosecurity assessment of Argentinian pig farms. Prev Vet Med. 2019;170:104637. doi: 10.1016/j.prevetmed.2019.02.012 31421498

[82] Anwar A, Malik M, Raees V, Anwar A. Role of Mass Media and Public Health Communications in the COVID-19 Pandemic. Cureus. 2020. 10.7759/cureus.10453 PMC7557800 33072461

[83] Gabarron E, Oyeyemi SO, Wynn R. COVID-19-related misinformation on social media: a systematic review. Bull World Health Organ. 2021;99(6):455–63. 34108756 10.2471/BLT.20.276782 PMC8164188

