# Supplementary material for: A socio-ecological approach to the determinants of animal health management: A scoping review
Source: PLoS One. 2026 Mar 20;21(3):e0344746. doi: 10.1371/journal.pone.0344746 (PMC13004347; doi:10.1371/journal.pone.0344746)
Supplement: S1 Table — (DOCX) [file pone.0344746.s001.docx]

**S1 Table. Keywords selected for articles search**

| **Keywords** |
| --- |
| Control |
| Policy |
| Measure |
| Crisis |
| Slaughter |
| Vaccination |
| Ban |
| Embargo |
| Containment |
| Monitoring |
| Prevention |
| Biosecurity |
| Hygiene |
| Cull |
| Infectious disease |
| Influenza |
| Bovine tuberculosis |
| BTB |
| FMD |
| Swine fever |
| Rabies |
| Blue tongue |
| Animal |
| Poultry |
| Bovine |
| Pork |
| Chicken |
| Pig |
| Cat |
| Dog |
| Horse |
