# Supplementary material for: A socio-ecological approach to the determinants of animal health management: A scoping review
Source: PLoS One. 2026 Mar 20;21(3):e0344746. doi: 10.1371/journal.pone.0344746 (PMC13004347; doi:10.1371/journal.pone.0344746)
Supplement: S2 Table — (DOCX) [file pone.0344746.s002.docx]

**S2 Table. Search engines and queries**

| Website | Scopus |
| --- | --- |
| Date of search | 2025-04-29 |
| Query | (TITLE-ABS-KEY(control OR polic* OR measure OR crisis OR slaughter OR vaccination OR ban OR embargo OR containment OR monitoring OR prevention OR biosecurity OR hygiene OR cull) AND TITLE-ABS-KEY((infectious W/0 disease) OR influenza OR (bovine W/0 tuberculosis) OR BTB OR FMD OR (swine W/0 fever) OR rabies OR (blue W/0 tongue)) AND TITLE-ABS-KEY(animal OR poultry OR bovine OR pork OR chicken OR pig OR cat OR dog OR horse)) AND ( LIMIT-TO ( SUBJAREA,"VETE" ) OR LIMIT-TO ( SUBJAREA,"AGRI" ) OR LIMIT-TO ( SUBJAREA,"MULT" ) OR LIMIT-TO ( SUBJAREA,"SOCI" ) OR LIMIT-TO ( SUBJAREA,"ARTS" ) OR LIMIT-TO ( SUBJAREA,"ECON" ) OR LIMIT-TO ( SUBJAREA,"HEAL" ) OR LIMIT-TO ( SUBJAREA,"BUSI" ) OR LIMIT-TO ( SUBJAREA,"DECI" ) OR LIMIT-TO ( SUBJAREA,"PSYC" ) ) AND ( LIMIT-TO ( DOCTYPE,"ar" ) ) AND ( LIMIT-TO ( PUBYEAR,2024) OR LIMIT-TO ( PUBYEAR,2023) OR LIMIT-TO ( PUBYEAR,2022) OR LIMIT-TO ( PUBYEAR,2021) OR LIMIT-TO ( PUBYEAR,2020) OR LIMIT-TO ( PUBYEAR,2019) OR LIMIT-TO ( PUBYEAR,2018) OR LIMIT-TO ( PUBYEAR,2017) OR LIMIT-TO ( PUBYEAR,2016) OR LIMIT-TO ( PUBYEAR,2015) OR LIMIT-TO ( PUBYEAR,2014) OR LIMIT-TO ( PUBYEAR,2013) OR LIMIT-TO ( PUBYEAR,2012) OR LIMIT-TO ( PUBYEAR,2011) OR LIMIT-TO ( PUBYEAR,2010)) |
| Limits | 2010 to 2025 |
| Number of articles | 13,673 |

| Website | Web of Sciences Core Collection |
| --- | --- |
| Date of search | 2025-04-29 |
| Query | (TS = (control) OR TS = (polic*) OR TS = (crisis) OR TS = (measure) OR TS = (slaughter) OR TS = (vaccination) OR TS = (ban) OR TS = (embargo) OR TS = (containment) OR TS =(monitoring) OR TS= (prevention) OR TS = (biosecurity) OR TS = (hygiene) OR TS = (cull))  AND (TS = ("infectious disease") OR TS =(influenza) OR TS = ("bovine tuberculosis") OR TS = (btb) OR TS =(fmd) OR TS = ("swine fever") OR TS = (rabies) OR TS = ("blue tongue"))  AND (TS = (animal) OR TS = (poultry) OR TS = (bovine) OR TS = (pork) OR TS = (chicken) OR TS = (pig) OR TS = (cat) OR TS = (dog) OR TS = (horse))  AND (DT=(Article)) |
| Limits | 2010-01-01 à 2025-04-29 |
| Number of articles | 9,010 |
