# Supplementary material for: A socio-ecological approach to the determinants of animal health management: A scoping review
Source: PLoS One. 2026 Mar 20;21(3):e0344746. doi: 10.1371/journal.pone.0344746 (PMC13004347; doi:10.1371/journal.pone.0344746)
Supplement: S3 Table — (DOCX) [file pone.0344746.s003.docx]

**S3 Table. Subject area selected by searching articles in Scopus**

| **Subject area Scopus** | **Abbreviation** |
| --- | --- |
| Veterinary | VETE |
| Agricultural and biological sciences | AGRI |
| Multidisciplinary | MULT |
| Social Science | SOCI |
| Arts and Humanities | ARTS |
| Economics, Econometrics, and Finance | ECON |
| Health Professions | HEAL |
| Business, Management, and Accounting | BUSI |
| Decision Science | DECI |
| Psychology | PSYC |
