# Supplementary material for: A socio-ecological approach to the determinants of animal health management: A scoping review
Source: PLoS One. 2026 Mar 20;21(3):e0344746. doi: 10.1371/journal.pone.0344746 (PMC13004347; doi:10.1371/journal.pone.0344746)
Supplement: S4 Table — (DOCX) [file pone.0344746.s004.docx]

**S4 Table. Web of Science categories selected by searching articles in Web of Science**

| **Web of Science Categories** |
| --- |
| Veterinary Science |
| Infectious diseases |
| Multidisciplinary Sciences |
| Agricultural Dairy Animal Science |
| Biology |
| Agriculture multidisciplinary |
| Agriculture Economics Policy |
| Economics |
| Social Sciences |
| Interdisciplinary |
| Health Policy Services |
| Management |
| Business |
| Humanities multidisciplinary |
| Business Finance |
