# Supplementary material for: A socio-ecological approach to the determinants of animal health management: A scoping review
Source: PLoS One. 2026 Mar 20;21(3):e0344746. doi: 10.1371/journal.pone.0344746 (PMC13004347; doi:10.1371/journal.pone.0344746)
Supplement: S5 Table — (DOCX) [file pone.0344746.s005.docx]

**S5 Table. Summary of eligibility criteria for the selection of articles included in the review**

| Inclusion | Exclusion |
| --- | --- |
| Livestock (cattle, sheep, goats, poultry, horses, pigs, fish), pets and wild animals | Human health and human diseases |
| Animal diseases including zoonoses | Lack of information on the applicability of management measures, i.e. how the measures are implemented |
| Analysis of the types of measures and their implementation (interventions, practices or strategies implemented to prevent, control or eradicate animal diseases) | Clinical cases, co-occurrence analysis of several species |
| Studies based on empirical data and field measurements | Animal management (farms, markets, individuals) with no specific link to animal diseases |
| Regulations/laws: quarantine measures, isolation, notification of cases of disease, biosecurity, mass vaccination, testing and slaughter, restrictions on animal movements | Comparison of disease transmission rates, transmission routes |
| Monitoring, control and eradication programmes, emergency plans, biosecurity | Different vaccination strategies: comparing vaccines and their efficacy |
| Sustainability of management measures, long-term effectiveness | Risks associated with food products of animal origin, ante-mortem and post-mortem examinations for food safety |
| Ex-ante and ex-post impacts | Disease transmission routes, transmission cycle, transmission dynamics |
| With and without management measures | Study of the spread/dispersal of a disease through a commercial network, in a farm |
| Simulations of intervention strategies, with comparisons of economic costs for example | Virus detection, virological studies |
| General approach by disease or species = general management approach | Prevalence, seroprevalence and disease occurrence studies |
| Applicability factors for implementing interventions | Genomics, biology of vaccines, diagnostic tests, serology, biomolecular studies, development of new vaccines |
| Determinants for the implementation of animal disease management measures | Study of the source, origin or focus of a disease or epidemic |
| Implication des acteurs dans la mise en œuvre des mesures de gestion (vétérinaires, éleveurs, communautés, gestionnaires, politiques) | Technical aspects of diagnostic tests, vaccines and surveillance Study of disease transmission using simulation models, transmission rates in different contexts, simulation of disease dispersion under different scenarios |
| Representation of management measures by professionals or animal owners and behaviour of stakeholders in the presence of diseases | Distribution of diseases |
| Holistic approach (interactions between animal diseases, humans and the environment) | Ecology of animal species |
| Meta-analyses, management case studies, systematic reviews | Effectiveness of vaccines and diagnostic tests |
| Sound and valid methodology | Theoretical models without empirical validation or practical application |
