# Supplementary material for: A socio-ecological approach to the determinants of animal health management: A scoping review
Source: PLoS One. 2026 Mar 20;21(3):e0344746. doi: 10.1371/journal.pone.0344746 (PMC13004347; doi:10.1371/journal.pone.0344746)
Supplement: S6 Table — (DOCX) [file pone.0344746.s006.docx]

**S6 Table. Frequency of studies by year**

| Year | Frequency (%) |
| --- | --- |
| 2010 | 24 (4%) |
| 2011 | 30 (5%) |
| 2012 | 28 (5%) |
| 2013 | 27 (4%) |
| 2014 | 24 (4%) |
| 2015 | 25 (4%) |
| 2016 | 34 (6%) |
| 2017 | 41 (7%) |
| 2018 | 45 (8%) |
| 2019 | 34 (6%) |
| 2020 | 53 (9%) |
| 2021 | 50 (8%) |
| 2022 | 50 (8%) |
| 2023 | 37 (6%) |
| 2024 | 74 (12%) |
| 2025 | 17 (3%) |
