# Supplementary material for: A socio-ecological approach to the determinants of animal health management: A scoping review
Source: PLoS One. 2026 Mar 20;21(3):e0344746. doi: 10.1371/journal.pone.0344746 (PMC13004347; doi:10.1371/journal.pone.0344746)
Supplement: S7 Table — (DOCX) [file pone.0344746.s007.docx]

**S7 Table. Frequency of studies by country**

| Country | Frequency |
| --- | --- |
| UK | 41 (7%) |
| USA | 33 (6%) |
| Australia | 24 (4%) |
| Ethiopia | 21 (4%) |
| Nigeria | 19 (3%) |
| France | 18 (3%) |
| China | 17 (3%) |
| Tanzania | 17 (3%) |
| Vietnam | 15 (3%) |
| India | 13 (2%) |
| Uganda | 13 (2%) |
| Indonesia | 12 (2%) |
| Ireland | 12 (2%) |
| South Africa | 11 (2%) |
| Nepal | 10 (2%) |
| Thailand | 10 (2%) |
| Philippines | 10 (2%) |
| Bangladesh | 9 (1%) |
| Cambodia | 9 (1%) |
| Germany | 9 (1%) |
| Italy | 9 (1%) |
| Canada | 8 (1%) |
| Spain | 8 (1%) |
| Brazil | 7 (1%) |
| Egypt | 7 (1%) |
| Japan | 7 (1%) |
| Netherlands | 7 (1%) |
| Sweden | 7 (1%) |
| Sri Lanka | 6 (1%) |
| South Korea | 6 (1%) |
| Danemark | 5 (0,8%) |
| Ghana | 5 (0,8%) |
| Kenya | 5 (0,8%) |
| New-Zeland | 5 (0,8%) |
| Switzerland | 5 (0,8%) |
| Zambia | 5 (0,8%) |
| Belgium | 4 (0,7%) |
| Cameroon | 4 (0,7%) |
| Chad | 4 (0,7%) |
| Laos | 4 (0,7%) |
| Madagascar | 4 (0,7%) |
| Serbia | 4 (0,7%) |
| Turkey | 4 (0,7%) |
| Pakistan | 4 (0,7%) |
| Bulgaria | 4 (0,7%) |
| Estonia | 3 (0,5%) |
| Iran | 3 (0,5%) |
| Java | 3 (0,5%) |
| Mongolia | 3 (0,5%) |
| Peru | 3 (0,5%) |
| Portugal | 3 (0,5%) |
| Argentina | 2 (0,3%) |
| Azerbaidjan | 2 (0,3%) |
| Benin | 2 (0,3%) |
| Finland | 2 (0,3%) |
| Haiti | 2 (0,3%) |
| Lithuania | 2 (0,3%) |
| Malawi | 2 (0,3%) |
| Mexico | 2 (0,3%) |
| Mozambique | 2 (0,3%) |
| Taiwan | 2 (0,3%) |
| Ukraine | 2 (0,3%) |
| Chile | 2 (0,3%) |
| Croatia | 2 (0,3%) |
| Congo | 2 (0,3%) |
| Russia | 2 (0,3%) |
| Albania | 1 (0,2%) |
| Antilles | 1 (0,2%) |
| Austria | 1 (0,2%) |
| Bhutan | 1 (0,2%) |
| Botswana | 1 (0,2%) |
| Burkina Faso | 1 (0,2%) |
| Burma | 1 (0,2%) |
| Grenada | 1 (0,2%) |
| Guinea | 1 (0,2%) |
| Hungary | 1 (0,2%) |
| Israel | 1 (0,2%) |
| Kazakhstan | 1 (0,2%) |
| Latvia | 1 (0,2%) |
| Malaysia | 1 (0,2%) |
| Morocco | 1 (0,2%) |
| Myanmar | 1 (0,2%) |
| Namibia | 1 (0,2%) |
| Norway | 1 (0,2%) |
| Poland | 1 (0,2%) |
| Sahel | 1 (0,2%) |
| Saudi Arabia | 1 (0,2%) |
| Senegal | 1 (0,2%) |
| Sierra Leone | 1 (0,2%) |
| Timor-Leste | 1 (0,2%) |
| Tunisia | 1 (0,2%) |
