# Supplementary material for: A socio-ecological approach to the determinants of animal health management: A scoping review
Source: PLoS One. 2026 Mar 20;21(3):e0344746. doi: 10.1371/journal.pone.0344746 (PMC13004347; doi:10.1371/journal.pone.0344746)
Supplement: S8 Table — (DOCX) [file pone.0344746.s008.docx]

**S8 Table. Characteristics of reviewed studies**

| Study Characteristics |  | Number of studies (percentage) |
| --- | --- | --- |
| Type of study | Qualitative | 187 (31%) |
|  | Survey | 122 (21%) |
|  | Quantitative | 125 (21%) |
|  | Evaluation | 44 (7%) |
|  | Modelisation | 30 (5%) |
|  | Review | 13 (2%) |
|  | Quantitative and qualitative | 49 (8%) |
|  | Narrative | 6 (1%) |
|  | Participative | 2 (0,3%) |
|  | Demographic analysis | 1 (0,2%) |
|  | Call to action | 1 (0,2%) |
|  |  |  |
| Disciplines | Epidemiology | 159 (27%) |
|  | Social sciences | 192 (32%) |
|  | Economics | 114 (19%) |
|  | Life sciences | 73 (12%) |
|  | Psychology | 22 (4%) |
|  | Sociology | 14 (2%) |
|  | Socio-economics | 12 (2%) |
|  | Public Health | 6 (1%) |
|  | Politics | 4 (0.7%) |
|  | Socio-psychology | 3 (0.5%) |
|  | Mathematics | 2 (0.3%) |
|  | Public policy | 4 (0.7%) |
|  | Anthropology | 1 (0.2%) |
|  |  |  |
| Level of analysis | National | 144 (24%) |
|  | Farm | 91 (15%) |
|  | Region | 104 (17%) |
|  | Local | 93 (16%) |
|  | Individual | 45 (8%) |
|  | Population | 25 (4%) |
|  | Sector | 22 (4%) |
|  | Global | 21 (3%) |
|  | International | 12 (2%) |
|  | Community | 11 (2%) |
|  | Subnational | 8 (1%) |
|  | Herd | 6 (1%) |
|  | City | 4 (0.7%) |
|  | Supranational | 4 (0.7%) |
|  | Market | 3 (0.5%) |
|  | Continental | 1 (0.2%) |
|  | Industry | 1 (0.2%) |
|  | Supply chain | 1 (0.2%) |
|  | Slaughterhouse | 1 (0.2%) |
|  |  |  |
| Situation | Routine | 395 (67%) |
|  | Crisis | 124 (21%) |
|  | Routine and crisis | 17 (3%) |
|  |  |  |
| Animal disease | Rabies | 146 (25%) |
|  | Avian influenza | 99 (17%) |
|  | Bovine tuberculosis | 68 (11%) |
|  | FMD | 63 (11%) |
|  | African swine fever | 49 (8%) |
|  | Infectious diseases | 47 (8%) |
|  | Equine influenza | 11 (2%) |
|  | Classical swine fever | 12 (2%) |
|  | Zoonosis | 16 (3%) |
|  | Johne’s disease | 5 (0,8%) |
|  | Influenza | 4 (0,7%) |
|  | Cysticercosis | 2 (0,3%) |
|  | Brucellosis | 6 (1%) |
|  | Bovine viral diarrhoea | 4 (0,7%) |
|  | Hendra virus | 2 (0,3%) |
|  | Salmonellosis | 4 (0,7%) |
|  | Japanese encephalitis | 1 (0,2%) |
|  | American foulbrood disease | 1 (0,2%) |
|  | Bluetongue | 1 (0,2%) |
|  | Bovine leucosis | 1 (0,2%) |
|  | Bovine brucellosis | 1 (0,3%) |
|  | Anthrax | 1 (0,2%) |
|  | Tularemia | 1 (0,2%) |
|  | Crimean-Congo haemorrhagic fever | 1 (0,2%) |
|  | Cystic echinococcosis | 2 (0,2%) |
|  | Toxoplasmosis | 1 (0,2%) |
|  | Contagious bovine pleuropneumonia | 1 (0,2%) |
|  | Endemic disease | 1 (0,2%) |
|  | Epizootic lymphangitis | 1 (0,2%) |
|  | Exotic diseases | 2 (0,3%) |
|  | Glanders | 1 (0,2%) |
|  | Equine diseases | 1 (0,2%) |
|  | Foodborne diseases | 1 (0,2%) |
|  | Infectious Laryngotracheitis | 1 (0,2%) |
|  | Leihsmaniosis | 1 (0,2%) |
|  | Lumpy skin disease | 1 (0,2%) |
|  | Mites | 1 (0,2%) |
|  | Neosporosis | 1 (0,2%) |
|  | Newcastle disease | 3 (0,5%) |
|  | Pancreatic disease and infectious salmon anemia | 1 (0,2%) |
|  | Porcine reproductive and respiratory syndrome | 2 (0,3%) |
|  | Swine influenza | 1 (0,2%) |
|  | Enzootic pneumonia | 1 (0,2%) |
|  | Geohelminthiasis | 1 (0,2%) |
|  | Strangles | 1 (0,2%) |
|  | Swine fever | 3 (0,5%) |
|  | Transmissible spongiform encephalopathy | 1 (0,2%) |
|  | Transboundary diseases | 3 (0,5%) |
|  |  |  |
| Species | Cattle | 140 (24%) |
|  | Dog | 129 (22%) |
|  | Poultry | 117 (20%) |
|  | Pig | 97 (16%) |
|  | Equine | 23 (4%) |
|  | Livestock | 20 (3%) |
|  | Wild boar | 15 (2%) |
|  | Badgers | 9 (1%) |
|  | Fox | 4 (0,7%) |
|  | Bats | 2 (0,3%) |
|  | Sheep | 9 (1%) |
|  | Human | 7 (1%) |
|  | Ruminants | 6 (1%) |
|  | Wildlife | 9 (1%) |
|  | Bee | 2 (0,3%) |
|  | Buffalo | 2 (0,3%) |
|  | Cat | 5 (0,8%) |
|  | Donkeys | 1 (0,2%) |
|  | Mules | 1 (0,2%) |
|  | Goat | 4 (0,7%) |
|  | Mussels | 1 (0,2%) |
|  | Ostriches | 1 (0,2%) |
|  | Raccoon | 2 (0,3%) |
|  | Small ruminants | 1 (0,2%) |
|  | Wild birds | 1 (0,2%) |
|  | Wild dog | 1 (0,2%) |
|  | Wild pigs | 1 (0,2%) |
|  | Domestic animals | 1 (0,2%) |
|  | All species | 1 (0,2%) |
|  | Duck | 1 (0,2%) |
