# Supplementary material for: A socio-ecological approach to the determinants of animal health management: A scoping review
Source: PLoS One. 2026 Mar 20;21(3):e0344746. doi: 10.1371/journal.pone.0344746 (PMC13004347; doi:10.1371/journal.pone.0344746)
Supplement: S9 Table — (DOCX) [file pone.0344746.s009.docx]

**S9 Table. Representation of management measures**

| Measures | Frequency | Percentage |
| --- | --- | --- |
| Vaccination | 234 | 39% |
| Biosecurity | 191 | 32% |
| Surveillance | 133 | 22% |
| Control | 73 | 12% |
| Culling | 56 | 9% |
| Prevention | 54 | 9% |
| Education | 27 | 4% |
| Eradication | 12 | 2% |
| Diagnostic | 12 | 2% |
| Awareness | 11 | 2% |
| Quarantine | 7 | 1% |
| Prophylaxis | 6 | 1% |
| Treatment | 3 | 0,5% |
| Ban | 2 | 0,3% |
| PEP | 2 | 0,3% |
| Traceability | 1 | 0,2% |
| Elimination | 1 | 0,2% |
| Movements restrictions | 1 | 0,2% |
| Isolation | 1 | 0,2% |
| Deworming | 1 | 0,2% |
| Financial compensation | 1 | 0,2% |
| Stamping-out | 1 | 0,2% |
| Trade restrictions | 1 | 0,2% |
| Sterilisation | 1 | 0,2% |
| Contraception | 1 | 0,2% |
| Euthanasia | 1 | 0,2% |
