# Supplementary material for: A socio-ecological approach to the determinants of animal health management: A scoping review
Source: PLoS One. 2026 Mar 20;21(3):e0344746. doi: 10.1371/journal.pone.0344746 (PMC13004347; doi:10.1371/journal.pone.0344746)
Supplement: S10 Table — (DOCX) [file pone.0344746.s010.docx]

**S10 Table. Actors involved in animal diseases management**

| Actors | Frequency | Percentage |  |
| --- | --- | --- | --- |
| Farmers | 301 | 51% |  |
| Citizens | 67 | 11% |  |
| Vets | 64 | 11% |  |
| Dog owners | 48 | 8% |  |
| Public authorities | 31 | 5% |  |
| Stakeholders | 28 | 5% |  |
| Government | 21 | 3% |  |
| Decision-makers | 17 | 3% |  |
| Authorities | 14 | 2% |  |
| State | 14 | 2% |  |
| Hunters | 12 | 2% |  |
| Sector | 9 | 1% |  |
| Health workers | 8 | 1% |  |
| Animal owners | 7 | 1% |  |
| Local authorities | 6 | 1% |  |
| Equine owners | 6 | 1% |  |
| Industry | 6 | 1% |  |
| Owners | 5 | 0,8% |  |
| General public | 5 | 0,8% |  |
| FAO, OIE, WHO | 3 | 0,5% |  |
| Farm staff | 3 | 0,5% |  |
| Technicians | 3 | 0,5% |  |
| Local laboratory | 3 | 0,5% |  |
| Slaughterhouse operators | 3 | 0,5% |  |
| Health authorities | 3 | 0,5% |  |
| Local communities | 3 | 0,5% |  |
| Professionals | 3 | 0,5% |  |
| Public and private actors | 3 | 0,5% |  |
| Health professionals | 2 | 0,3% |  |
| NGOs | 2 | 0,3% |  |
| Coordinator | 2 | 0,3% |  |
| Conservationists | 2 | 0,3% |  |
| Wildlife organisations | 2 | 0,3% |  |
| Veterinary services | 2 | 0,3% |  |
| International institutions | 2 | 0,3% |  |
| Butchers | 2 | 0,3% |  |
| Suppliers | 2 | 0,3% |  |
| Traders | 2 | 0,3% |  |
| Public agents | 2 | 0,3% |  |
| Policy-makers | 2 | 0,3% |  |
| Students | 2 | 0,3% |  |
| Abatoir workers | 1 | 0,2% |  |
| Drivers | 1 | 0,2% |  |
| Experts in control | 1 | 0,2% |  |
| Agricultural authorities | 1 | 0,2% |  |
| National reference laboratory | 1 | 0,2% |  |
| Control unit | 1 | 0,2% |  |
| Forest wardens | 1 | 0,2% |  |
| Public administration | 1 | 0,2% |  |
| National agencies | 1 | 0,2% |  |
| Scientists | 1 | 0,2% |  |
| Distributors | 1 | 0,2% |  |
| Carriers | 1 | 0,2% |  |
| Exporters | 1 | 0,2% |  |
| GDS | 1 | 0,2% |  |
| Transporters | 1 | 0,2% |  |
| Internation community | 1 | 0,2% |  |
| Mule drivers | 1 | 0,2% |  |
| Services providers | 1 | 0,2% |  |
| OIE members | 1 | 0,2% |  |
| Partner countries | 1 | 0,2% |  |
| Pet owners | 1 | 0,2% |  |
| Sellers | 1 | 0,2% |  |
| Private investors | 1 | 0,2% |  |
| Jokers | 1 | 0,2% |  |
| Consummers | 1 | 0,2% |  |
| Supply chain | 1 | 0,2% |  |
| Surveillance laboratory | 1 | 0,2% |  |
| Private advisors | 1 | 0,2% |  |
| Epidemiologists and pathologists | 1 | 0,2% |  |
| Administrative staff | 1 | 0,2% |  |
| Victims of dog bites | 1 | 0,2% |  |
| Workers | 1 | 0,2% |  |
