# Supplementary material for: A socio-ecological approach to the determinants of animal health management: A scoping review
Source: PLoS One. 2026 Mar 20;21(3):e0344746. doi: 10.1371/journal.pone.0344746 (PMC13004347; doi:10.1371/journal.pone.0344746)
Supplement: S11 Table — (DOCX) [file pone.0344746.s011.docx]

**S11 Table. Frequency of applicability factors**

| **Factors** | **Frequency (%)** |
| --- | --- |
| Individual and socio-cognitive | 332 (56%) |
| Economic | 271(46%) |
| Socio-political and institutional | 251(42%) |
| Organisational and professional | 195 (33%) |
| Infrastructure | 179 (30%) |
