# Supplementary material for: A socio-ecological approach to the determinants of animal health management: A scoping review
Source: PLoS One. 2026 Mar 20;21(3):e0344746. doi: 10.1371/journal.pone.0344746 (PMC13004347; doi:10.1371/journal.pone.0344746)
Supplement: S12 Table — (DOCX) [file pone.0344746.s012.docx]

**S12 Table. Most frequent word for each applicability factors**

| Factor | Word | Frequency |
| --- | --- | --- |
| Economic | cost | 157 |
|  | economic | 63 |
|  | financial | 34 |
|  | vaccination | 32 |
|  | impact | 30 |
|  | measure | 29 |
|  | compensation | 26 |
|  | benefit | 22 |
|  | lack | 20 |
|  | resource | 19 |
| Individual and socio-cognitive | knowledge | 122 |
|  | representation | 87 |
|  | level | 83 |
|  | education | 67 |
|  | lack | 66 |
|  | farmer | 56 |
|  | awareness | 55 |
|  | behaviour | 46 |
|  | dog | 44 |
|  | age | 36 |
| Infrastructure | livestock | 64 |
|  | system | 63 |
|  | lack | 26 |
|  | farm | 23 |
|  | animal | 22 |
|  | vaccination | 20 |
|  | access | 18 |
|  | movement | 15 |
|  | infrastructure | 14 |
|  | area | 13 |
| Organisational and professional | lack | 59 |
|  | vaccination | 45 |
|  | veterinary | 40 |
|  | information | 29 |
|  | service | 24 |
|  | train | 22 |
|  | access | 20 |
|  | vaccine | 18 |
|  | availability | 17 |
|  | poor | 17 |
| Socio-political and institutional | lack | 63 |
|  | measure | 50 |
|  | political | 42 |
|  | coordination | 38 |
|  | government | 33 |
|  | farmer | 29 |
|  | communication | 28 |
|  | information | 27 |
|  | control | 24 |
|  | community | 23 |
