# Supplementary figures and images for: A socio-ecological approach to the determinants of animal health management: A scoping review
Source: PLoS One. 2026 Mar 20;21(3):e0344746. doi: 10.1371/journal.pone.0344746 (PMC13004347; doi:10.1371/journal.pone.0344746)

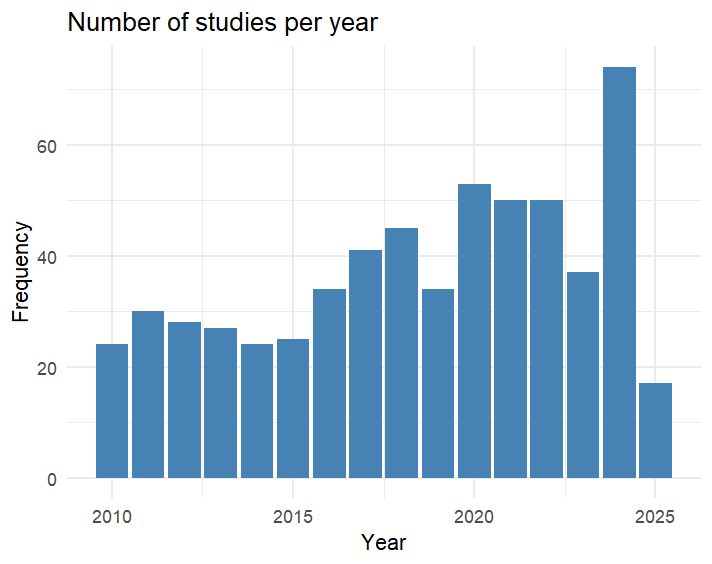


**S1 Fig. Occurrence of reviewed studies by year of publication**

Supplement: S1 Fig — (DOCX) [file pone.0344746.s015.docx]

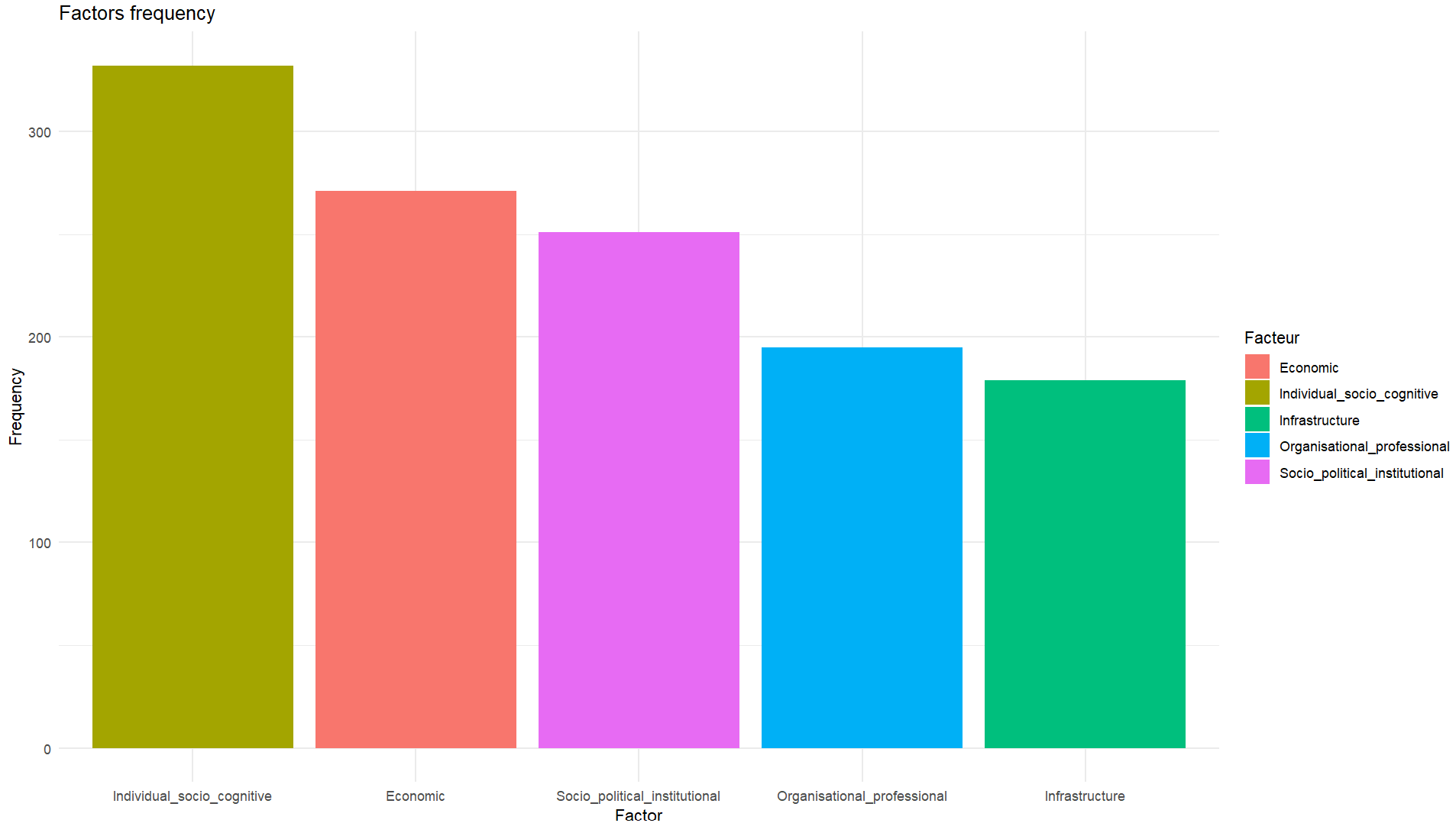


**S4 Fig. Representation of applicability factors**

Supplement: S4 Fig — (DOCX) [file pone.0344746.s018.docx]
