# Supplementary material for: A socio-ecological approach to the determinants of animal health management: A scoping review
Source: PLoS One. 2026 Mar 20;21(3):e0344746. doi: 10.1371/journal.pone.0344746 (PMC13004347; doi:10.1371/journal.pone.0344746)
Supplement: S3 Fig — (DOCX) [file pone.0344746.s017.docx]

**S3 Fig. Top 10 of actors involved in animal diseases management**
